# Temporal trends in breast cancer presentation in the third world

**DOI:** 10.1186/1756-9966-27-17

**Published:** 2008-07-11

**Authors:** Stanley NC Anyanwu

**Affiliations:** 1 Department of Surgery, Nnamdi Azikiwe University Teaching Hospital, Nnewi, Nigeria

## Abstract

**Background:**

Third world breast cancer is characterized by late presentation, occurrence at relatively young ages and dismal mortality. This poor outcome has encouraged patients to patronize quacks and alternative healers. Public control measures have targeted mainly public education and provision of screening facilities. Recent reports from the developed world indicate a high association with obesity, tobacco and alcohol, habits which though not currently very popular in the third world are nevertheless increasingly accepted.

**Methods:**

A prospective study initiated in 1985 for all breast cancer patients attending 4 hospitals located in the Eastern Nigeria heartland where the author practiced. On attendance to hospital detailed epidemiological data including social habits were collected from patients.

**Results:**

Reports from our first series [1987–97] showed some improvement in terms of earlier presentation compared to a historical control of earlier reports from the sub-region. Reports from the present study showed that this improvement has not been maintained probably as a result of diversion of public health campaign finances to HIV/AIDS. However there is an increasing mean age of presentation due to a higher representation of above 70 years age group and a significant reduction in parity. Alcohol intake and smoking have remained at low levels among the patients.

**Conclusion:**

There is need to take another look at cancer public health campaign mechanisms in the face of competing demands from HIV. Public control measures should include among others teaching of Breast Self Examination [BSE] to patients, Clinical Breast Examination [CBE] to health workers and opportunistic CBE to all patients. Strenuous efforts should be made to break the vicious cycle of late presentation, poor treatment outcome and reluctance of patients to present to health facilities because of poor outcome.

## Background

Breast cancer is now the most common female malignancy world-wide with up to a million cases annually[1,2]. Areas of greatest incidence have been N. America and Europe with black Africa considered an area of low incidence[2]. Studies from Nigeria indicate breast cancer has recently overtaken cervical cancer as the commonest female malignancy in areas of Western and Eastern Nigeria[3]. In other parts of the world racial incidence rates have changed due from migration and adoption of 'Western' life-style [4-6]. General worldwide mortality rates have decreased as a result of earlier detection, more favourable stage distribution and improved management[7]. However while the decline is marked for younger patients, moderate for middle-aged, the rates in elderly women have shown some increase[8]. In the third world and in African-Americans breast cancer presents at advanced stages and with worse biologic behavior[9,10]. Reasons for late presentation are believed to be due to ignorance, superstition, self-denial, fear of mastectomy and unavailability of treatment facilities[7,10-12]. Recent approaches have targeted public health awareness campaigns to encourage earlier diagnosis. These public awareness started in earnest in Nigeria in mid-80's and were spear-headed by Nigeria Cancer Society and other agencies. However due to the current HIV/AIDS pandemic most public health campaign resources are being diverted to that sector. We established a breast unit in 1987 to study aspects of the disease in parts of Eastern Nigeria. Our initial experiences in first ten years showed some moderate improvement from earlier reports in terms of earlier presentation and reduced mortality rates[13]. We decided to present our later experiences to review aspects of disease bearing in mind increasing 'Westernization' of the society and the decrease in public awareness campaigns.

## Methods

All consecutive patients with breast disease referred to the authors practices at Nnamdi Azikiwe University Teaching Hospital, Nnewi [public patients] and Ace Specialist Hospital, Onitsha [private patients] all in Anambra State of Nigeria were enrolled. Information obtained included age, sex, marital status, educational and social status, menarche, menopause, lactation, social habits like smoking and alcohol intake, examination findings including obesity, tumour assessment and staging, histologic studies, treatment and outcome. Data were extracted for analysis including comparison with our initial experiences of 1987–97 and to other published data from Nigeria. Assessment of metastasis was limited to clinical examination, chest X-Rays, skeletal X-Rays and abdominal ultrasonography.

## Results

### Epidemiology

**Figure 1:**
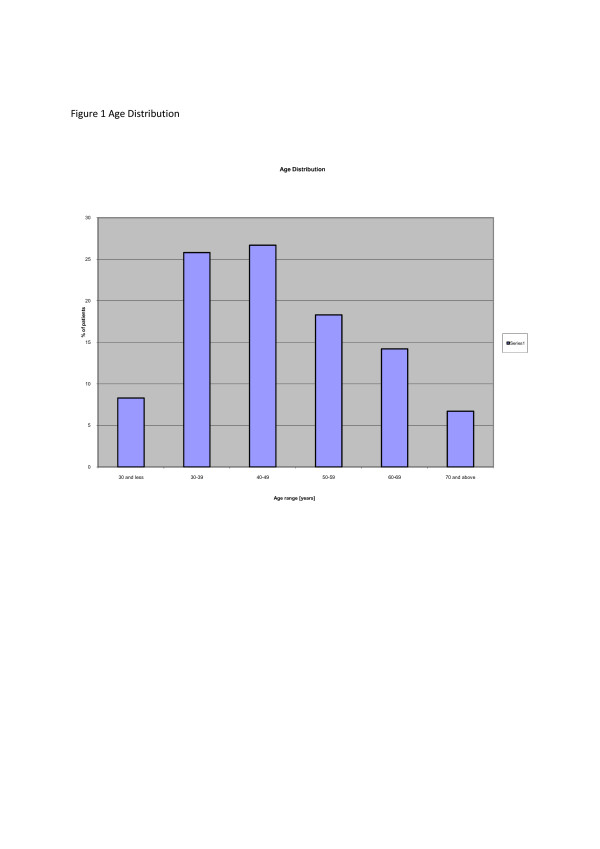
Age distribution.

During the period 1998–2005 [8 years] 179 new cases of primary female breast cancer were seen. The age range was 17 to 80 years with a mean of 46.85 years [SD 12.98, SEM 1.45]. The peak age ranges were 30–39 and 40–49. Less than 10% of patients were aged less than 30 years and more than 70 years respectively. The mean ages of private and p ublic hospital patients were 48.2 and 46.2 years respectively. The age distribution is shown in Fig. 1.

**Figure 2:**
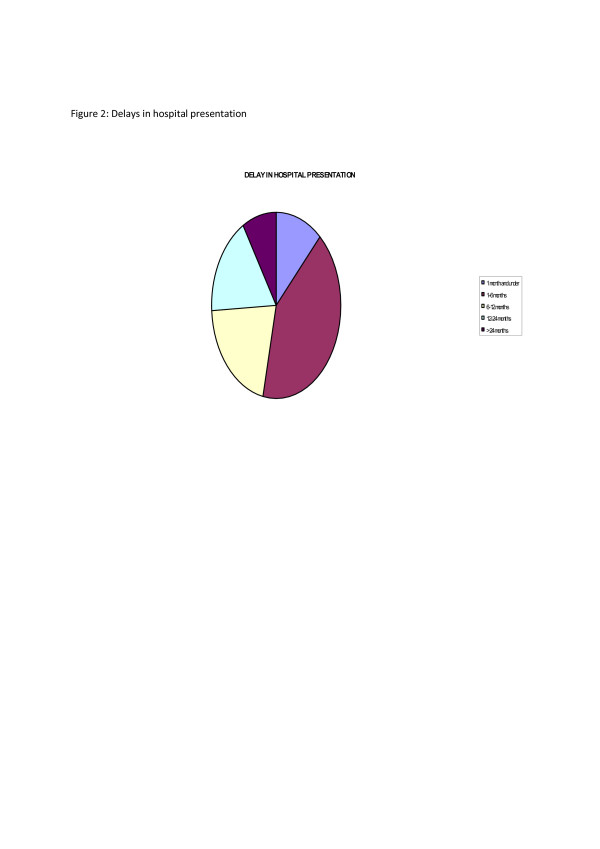
Delay in hospital presentation.

Delay from initial notice of symptoms to hospital presentation ranged from 5 days to 9 years. Twelve percent presented at 1 month and under, 41% between 1 and 6 months, 21% between 6 and 12 months, 17% between 12 and 24 months and 9% at more than 24 months as shown in Fig. 2.

Eighty-eight percent of patients were married, separated, widowed or divorced at time of presentation. Mean age at menarche was 13.67 years [SD 1.61, SEM 0.15], 43% were menopausal at time of initial consultation; mean age at menopause was 46.34 years [SD 4.73, SEM 0.43]. Mean parity was 4.28 [SD 3.00, SEM 0.28], range 0 to 10; 17% were nulliparous. All parous patients had breastfed their babies, the total duration ranging from 5 to 216 months. Ninety-two percent of the patients had never used oral contraceptives, of the few that used, usage was occasional in most.

Smoking among patients was not admitted to while alcohol intake was limited to social [less than a drink monthly] drinking in less than 15% of the patients.

Ten percent gave a positive family history of breast cancer with the sister more often affected followed by cousins. Past history of breast cancer was obtained in 2 patients, the longest haven been treated 10 years previously. Sixty percent of the patients had a minimum of high school education.

### Clinical findings

**Figure 3:**
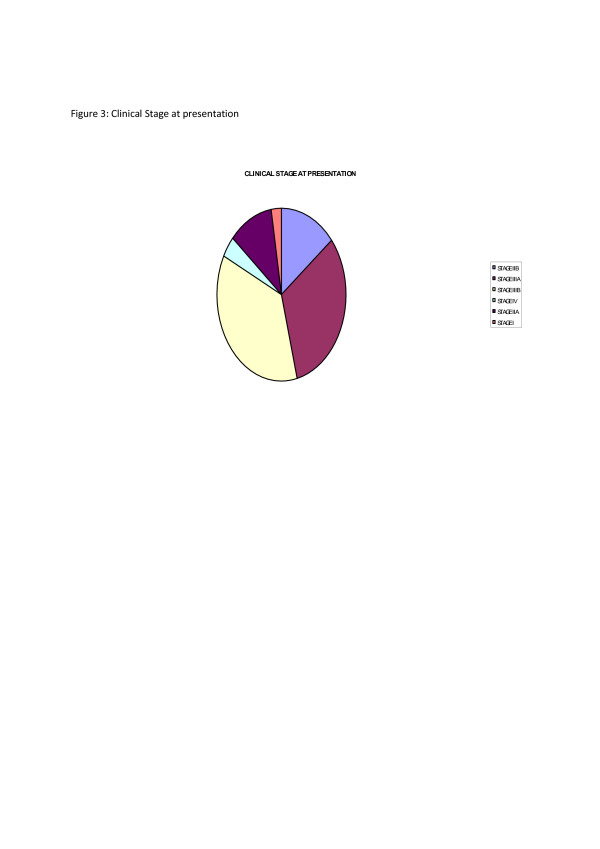
Clinical stage at presentation.

Forty nine percent each occurred in left and right breasts respectively while 2% were bilateral. Three percent of the patients presented with Stage I, 11% in Stage IIA, 14% with Stage IIB, 32% with Stage IIIA, 36% with Stage IIIB and 4% with obvious distant metastasis in Stage IV as shown in Fig. 3. Of course under-staging is possible as only clinical methods with supportive radiology was utilized. Clinical lymph node positivity was noted in 72%. There were no mammographically diagnosed cases.

**Figure 4:**
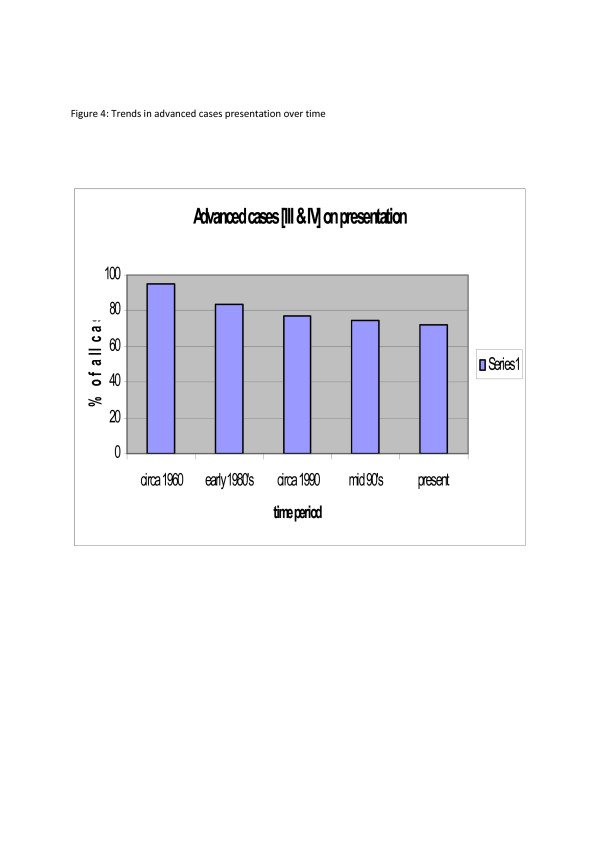
Advanced cases on presentation.

Elderly patients and those from poorer social classes presented at later stages. Whereas public health education has resulting in significant down-staging of breast cancer in other parts of the world, the results from our study when compared to previous studies from the same environment show very marginal improvement (Fig. 4).

The commonest histologic diagnosis was infiltrating ductal carcinoma in 80% followed by intraductal carcinoma in 7%, mucinous in 4%, lobular in 2% and others including a case each of malignant phylloides tumour and squamous cell carcinoma.

## Discussion

Compared to our previous series[13] there has been a significant increase in hospital incidence of breast cancer. This is in line with other reports from the third world and from global incidence studies[2,4,5]. However whether this increase is due to increasing awareness as suggested by some workers or reflects a global trend will need population based studies to elucidate. The only population based study in Eastern Nigeria[14] indicate a prevalence rate of 0.22% in nuns aged 20 to 68 years. The mean age at presentation has shown some [non-significant] increase from 44 years in our previous series to 46.9 years, a figure that is approaching the Asian and Middle Eastern population mean of 50[15,16] but still a decade younger than the Western mean. Our findings indicate that the slight increase in mean age is due more to a higher representation of patients aged above 70 than in previous studies. This higher representation of above 70 age group is probably due to hospitals being nearer most communities or by better care by their children.

Significant delays in presentation still abound with only 12% presenting within 1 month of noticing symptoms. This shows a marginal increase from 7% noted in our previous study[13]. This late presentation is universal among patients in the third world including Asian and Arab countries. Reasons adduced have included long distances to hospital, lack of awareness, fear of the consequences, strong belief in traditional medicine, religious charlatans, poverty, poor education and fear and denial. Improvement in public health awareness as recommended by others will surely help. However most funds by Governments and donor agencies to sub-Saharan Africa are targeted towards HIV prevention campaigns. Probably adding talks on cancer during the talks as part of general health measures may assist. Indeed poor funding has severely limited the activities of the Nigerian Cancer Society and other societies involved in cancer control. Quite worrisome is the observation that a majority of the patients had attended a health facility in the preceding 6 months without anybody examining their breasts. We cannot but concur with Smith et al[11] that training of relevant staff in clinical evaluation of both symptomatic and asymptomatic women, opportunistic screening and organized trials of BSE will help achieve earlier detection in resource – poor communities. Oluwole et al[17] achieved a marked reduction in mortality and better conservation after introduction of screening in a poor urban community of Harlem in North America.

Coupled with delays in presentation is non-improvement in the clinical stage at presentation despite the above 60% literacy level in the society when compared to our previous studies[9,13,18,19]. With more than 70% presenting with Stages III and IV and 72% with lymph node positivity curative therapies become elusive. This gloomy picture reinforces the negative perception of the disease in the public and maintains the vicious cycle associated with cancer in the third world. Unlike parts of Asia[15] where a small number of cases present early with mammographic diagnosis, such was not the case in this study. While mammograms are available in some centres, awareness and necessary skills in interpretation are lacking.

Current studies confirm earlier reports of falling age at menarche, falling parity and increasing age at menopause[13]. These factors have been implicated in breast cancer increase in other societies. Although the risk associated with oral contraceptives and abortion are still the subjects of considerable dispute[20] both are uncommon in the breast cancer population of Nigeria. On a happier note however other risk factors like alcohol intake, tobacco and obesity are still uncommon in the breast cancer population in this study.

## Conclusion

Breast cancer still presents late in the third world with a widening of the 'mortality gap' Public health enlightenment measures for cancer seem to have fallen victim to the global war on HIV/AIDS. Radical policies including public campaigns with emphasis on BSE and CBE should be encouraged, while deleterious societal mindsets should be tackled. It may be wise to set targets for earlier detection to encourage curative and breast conservation measures.

## Competing interests

The authors declare that they have no competing interests.

## Authors' contributions

SNC: design, collection of data, preparation of manuscript.
